# Plant genotype-specific archaeal and bacterial endophytes but similar *Bacillus* antagonists colonize Mediterranean olive trees

**DOI:** 10.3389/fmicb.2015.00138

**Published:** 2015-03-03

**Authors:** Henry Müller, Christian Berg, Blanca B. Landa, Anna Auerbach, Christine Moissl-Eichinger, Gabriele Berg

**Affiliations:** ^1^Institute of Environmental Biotechnology, Graz University of TechnologyGraz, Austria; ^2^Botanical Garden, Institute of Plant Sciences, University of GrazGraz, Austria; ^3^Institute for Sustainable Agriculture, Spanish National Research CouncilCórdoba, Spain; ^4^Department for Microbiology and Archaea Center, University of RegensburgRegensburg, Germany; ^5^Department of Internal Medicine, Medical University of GrazGraz, Austria; ^6^BioTechMed, GrazAustria

**Keywords:** endophytes, *Olea europaea*, Archaea, antagonistic bacteria, *Verticillium dahliae*

## Abstract

Endophytes have an intimate and often symbiotic interaction with their hosts. Less is known about the composition and function of endophytes in trees. In order to evaluate our hypothesis that plant genotype and origin have a strong impact on both, endophytes of leaves from 10 *Olea europaea* L. cultivars from the Mediterranean basin growing at a single agricultural site in Spain and from nine wild olive trees located in natural habitats in Greece, Cyprus, and on Madeira Island were studied. The composition of the bacterial endophytic communities as revealed by 16S rRNA gene amplicon sequencing and the subsequent PCoA analysis showed a strong correlation to the plant genotypes. The bacterial distribution patterns were congruent with the plant origins in “Eastern” and “Western” areas of the Mediterranean basin. Subsequently, the endophytic microbiome of wild olives was shown to be closely related to those of cultivated olives of the corresponding geographic origins. The olive leaf endosphere harbored mostly *Proteobacteria*, followed by *Firmicutes, Actinobacteria,* and *Bacteroidetes*. The detection of a high portion of archaeal taxa belonging to the phyla *Thaumarchaeota, Crenarchaeota,* and *Euryarchaeota* in the amplicon libraries was an unexpected discovery, which was confirmed by quantitative real-time PCR revealing an archaeal portion of up to 35.8%. Although the function of these Archaea for their host plant remains speculative, this finding suggests a significant relevance of archaeal endophytes for plant–microbe interactions. In addition, the antagonistic potential of culturable endophytes was determined; all isolates with antagonistic activity against the olive-pathogenic fungus *Verticillium dahliae* Kleb. belong to *Bacillus amyloliquefaciens*. In contrast to the specific global structural diversity, BOX-fingerprints of the antagonistic *Bacillus* isolates were highly similar and independent of the olive genotype from which they were isolated.

## INTRODUCTION

Olive trees (*Olea europaea* L.) represent one of the most ancient domestic plants, which have characterized the Mediterranean landscape since ancient times (Zohary and Spiegel-Roy, 1975). Olives originated from Asia and spread from Iran, Syria, and Palestine to the rest of the Mediterranean basin 6,000 years ago (Breton et al., 2008, 2009). The species *O. europaea* L. is classified as wild, referred to as oleaster (subsp. *europaea* var. *sylvestris*), and as cultivated (subsp. *europaea* var. *europaea*) types (Green, 2002). The domestication and breeding history of olive trees has not been fully described to date. Ancestral wild gene pools from three long-term refugia (the Near East, the Aegean area, and the Strait of Gibraltar) have provided the essential foundations for cultivated olive breeding (Besnard et al., 2013). At present, a long list of genotypes cultivated in the Mediterranean basin exists (Lumaret and Quazzani, 2001; Díaz et al., 2006; Díez et al., 2011). According to their gene pools olive cultivars can be divided into three main groups related to the region of origin “Eastern,” “Central,” and “Western” (Haouane et al., 2011). Today, olive trees represent one of the most important oil crops world-wide, delivering monounsaturated fatty acid and antioxidant-containing olive oil, which serves as the major fatty component of the Mediterranean diet. In 2013, on an area of 10.2 Mio ha 20.3 Mio t of olives was harvested world-wide and showed in the last few years a strong upward trend (FAOSTAT, 2014). However, olive production in the Mediterranean region is affected by several diseases. *Verticillium* wilt, caused by *Verticillium dahliae* Kleb., is currently the most devastating disease correlated with low yield and high rates of tree loss (López-Escudero and Mercado-Blanco, 2011). Since no resistant varieties and effective fungicides exist, biological control using the naturally occurring antagonistic potential against pathogens is a potentially viable and environmentally friendly alternative (Jiménez-Díaz et al., 2011). Although several successful example studies were published for the pathosystem olive-*Verticillium* (Prieto and Mercado-Blanco, 2008; Maldonado-González et al., 2013, 2015), inconsistent effects in the field are one hurdle along the path towards commercialization. Microbiome-based biocontrol strategies can solve these problems (Berg et al., 2013; Berg, 2014) but have not yet been established.

Endophytes that live inside plants do not cause harm to the plants and are characterized by an intimate interaction with their hosts (Hallmann et al., 1997; Hardoim et al., 2008; Reinhold-Hurek and Hurek, 2011). Endophytes with antagonistic activity against pathogens are promising candidates for biocontrol strategies against *Verticillium* because they colonize the same niche and can compete directly with the pathogen. The endophytic microbiome shows great diversity, which is influenced by the site and growth stage of the host plants as well as fulfilling important functions for its host including the promotion of plant growth, protection against biotic, and abiotic stress as well as the production of essential secondary metabolites (Ryan et al., 2008; Berg, 2009; Alavi et al., 2013). Although a large diversity of microorganisms can live endophytically, mainly bacteria, in particular *Alphaproteobacteria,* were identified as plant inhabitants (Bulgarelli et al., 2013). In contrast, much less is known about endophytic Archaea. Archaea represent the so-called third domain of life, and have only recently been described as important component of the moderate environment and the human microbiome (Probst et al., 2013). A few very recent publications have mentioned internal plant tissue colonization by members of the Archaea (Ma et al., 2013; Oliveira et al., 2013), but their distribution, significance, function, and activity remains unclear. In addition, the endophytic microbiota of trees has undergone less investigation and nothing is known about the associated microorganisms within olive trees. Our hypothesis has been that a positive identification of olive-associated endophytic communities depends on whether their patterns are found to correspond with their geographical origin. Because of the longevity and the high genetic variability of oleasters and cultivars, olives might have a specific but stable community of microbes over periods and ranges and should contain a high diversity of microbes, especially with antagonistic potential against *V. dahliae* (Aranda et al., 2011).

The objective of this study was to determine and compare the structure of endophytic microbiota of 10 *O. europaea* L. cultivars from the Mediterranean basin at one agricultural site in Spain and from nine wild olive trees located in natural habitats in Greece, Cyprus, and on Madeira Island by a set of molecular and isolation-dependent methods. Moreover, the study addressed the question what factors shape the endophytic microbiome and, in particular, whether the bacterial and archaeal communities reflects the different geographic origins of the investigated olive genotypes. The results will be used to reveal influences on the tree microbiome but also to develop successful biocontrol strategies against *Verticillium* wilt in olives.

**Table 1:** Sample designation, cultivar, and geographical origin used in this study.

Sample designation	Cultivar	Origin	Coordinates
SP3	Arbequino	Spain	37°56^′^26.62^′′^ N, 03°47^′^06.78^′′^ W
SP5	Ocal	Spain	37°56^′^26.62^′′^ N, 03°47^′^06.78^′′^ W
I2	Leccino	Italy	37°56^′^26.62^′′^ N, 03°47^′^06.78^′′^ W
GR1	Koroneiki	Greece	37°56^′^26.62^′′^ N, 03°47^′^06.78^′′^ W
GR2	Kalamata	Greece	37°56^′^26.62^′′^ N, 03°47^′^06.78^′′^ W
TUN1	Chétoni	Tunisia	37°56^′^26.62^′′^ N, 03°47^′^06.78^′′^ W
SI1	Trylia	Syria	37°56^′^26.62^′′^ N, 03°47^′^06.78^′′^ W
MO1	Picholine Marrocaine	Morocco	37°56^′^26.62^′′^ N, 03°47^′^06.78^′′^ W
PO1	Galega	Portugal	37°56^′^26.62^′′^ N, 03°47^′^06.78^′′^ W
FR1	Aglandau	France	37°56^′^26.62^′′^ N, 03°47^′^06.78^′′^ W
CY1	Wild	Cyprus	35°04^′^09.64^′′^ N, 32°18^′^00.28^′′^ E
CY2	Wild	Cyprus	35°05^′^01.13^′′^ N, 32°18^′^00.10^′′^ E
CY3	Wild	Cyprus	34°46^′^06.99^′′^ N, 32°54^′^08.96^′′^ E
CY4	Wild	Cyprus	34°46^′^41.94^′′^ N, 32° 54^′^53.76^′′^ E
GR(w)1	Wild	Greece	38°12^′^04.84^′′^ N, 22°06^′^05.22^′′^ E
GR(w)2	Wild	Greece	38°12^′^06.01^′′^ N, 22°06^′^01.38^′′^ E
GR(w)3	Wild	Greece	38°10^′^00.54^′′^ N, 22°06^′^05.88^′′^ E
GR(w)4	Wild	Greece	38°10^′^04.68^′′^ N, 22°06^′^36.42^′′^ E
M1	Wild	Madeira	32°44^′^13.37^′′^ N, 17°12^′^34.77^′′^ W

## MATERIALS AND METHODS

### SAMPLING STRATEGY

Cultivated and wild olive trees from different regions were sampled. The samples of the cultivated olives were taken at a single experimental orchard at the ‘Venta del Llano’ Research Station (IFAPA, Andalusia Regional Government) in Mengibar (Jaén province, southern Spain). The field was established 22 years ago using olive planting stocks of the same age of different cultivars of various Mediterranean origins (Palomares-Rius et al., 2012). From four trees of selected cultivars, vegetative branches and adherent leaves were sampled in May 2009 (**Table 1**). Always the terminal ends (10 cm) from four of the youngest branches around an individual tree were taken and pooled. For the sampling surface-disinfected gloves and scissors as well as sterile bags were used. Leaves and boughs of wild olives were collected from Cyprus (February, 2009), Greece (May, 2009), and Madeira (August, 2009). Samples from olive cultivars in Mengibar were chilled and processed within one day, whereas the material from wild olives were stored and transported under cooled condition until processing within at least four days.

### DNA EXTRACTION

For the isolation of microorganisms, 10 g of leaves and boughs from each composite sample of individual trees were surface-sterilized for 5 min using sodium hypochlorite (3% active chlorine) and washed three times with autoclaved water. After the addition of 5 mL of sterile water the samples were pestled and 2 mL of the suspension was transferred in a 2 mL tube, and centrifuged at 16.500 ×*g* for 15 min at 4°C. The supernatant was discarded and the pellet was stored at -21°C. Total DNA of bacterial and fungal consortia was extracted using the FastDNA®; Spin Kit for Soil (MP Biomedicals, Santa Ana, CA, USA) according to the manufacturer’s protocol.

### STRUCTURE OF ENDOPHYTIC BACTERIAL COMMUNITIES REVEALED BY ILLUMINA MiSeq AMPLICON SEQUENCING

To analyze the taxonomic composition of the endophytic bacterial communities an amplicon sequencing approach using Illumina’s MiSeq platform was applied for three biological replicates per studied cultivar or oleaster. The hypervariable V4 region of the 16S rRNA gene was amplified according to the protocol described by Caporaso et al. (2012) using the region specific primer pair 515f and 806r that included Illumina cell flow adaptors and sample-specific barcodes. The PCR reaction mixture (30 μl) contained 1x Taq&Go (MP Biomedicals, Illkirch, France), 0.25 mM of each primer and 1 μl of template DNA (94°C for 3 min, 32 cycles of 94°C for 45 s, 60°C for 1 min, 72°C for 18 s, and final elongation at 72°C for 10 min). Products from three independent PCR runs for each sample were pooled in equal volumes and purified by employing the Wizard SV Gel and PCR Clean-Up System (Promega, Madison, WI, USA). After spectrophotometrical measurement of DNA concentrations (Nanodrop 2000, Thermo Scientific, Wilmington, DE, USA) equimolar aliquots of all samples were combined for amplicon sequencing using Illumina MiSeq v2 (250 bp paired end) conducted by (LGC Genomics, Berlin, Germany). Raw sequencing data preparation included demultiplexing (CASAVA data analysis software, Illumina), clipping of sequencing adapters (TruSeq, Illumina), joining read pairs (FLASH 1.2.4, Magoc and Salzberg, 2011) with a minimum overlap of 10 bases and maximum mismatch density of 0.25, and sorting according to sample-specific barcodes. Prior to the next step, reads from biological replicates from each cultivar/oleaster were joined (Supplementary Table S1). Resulting reads were quality (Phred score ≥ 20) and length filtered (290–300 bp) using scripts provided by the open source software package QIIME 1.8.0 (). Chimeric sequences were discarded after *de novo* detection based on USEARCH 6.1 (Edgar et al., 2011). UCLUST algorithm using default parameters was applied to cluster remaining reads to operational taxonomic units (OTUs) at 97% similarity (Edgar, 2010) followed by taxonomic assignment of representative sequences by RDP naïve Bayesian rRNA classifier (Wang et al., 2007) based on the reference database Greengenes release gg_13_8_99 (DeSantis et al., 2006). Archaeal reads were additionally classified using Silva’s SINA aligner (Pruesse et al., 2012). Prior to further analysis all reads assigned to plant plastids (chloroplasts and mitochondria) were discarded from datasets. The number of sequences of each sample was normalized to the lowest number of read counts by randomly selecting subsets of sequences by a custom Perl script (10-times random sampling followed by subset formation). Principal Coordinate Analysis (PCoA) was performed to assess the beta diversity based on the calculation of the weighted normalized UniFrac distance matrix (Lozupone and Knight, 2005). The study is registered as NCBI BioProject PRJNA272855, the metadata for each sample are available at NCBI in the BioSample database (accession numbers SAMN03287521 – 33), and the sequence data are deposited in NCBI’s Short Read Archive (SRA) under accession numbers SRR1781607, SRR1781712, SRR1781720, SRR1781736, SRR1781767, SRR1781768, SRR1781984, SRR1781986, SRR1781987, SRR1781988, SRR1781989, SRR1781990, and SRR1782571.

### QUANTIFICATION OF ARCHAEA POPULATION BY QUANTITATIVE REAL-TIME PCR (qPCR)

Bacteria- and archaea-directed quantitative real-time PCR (qPCR) was performed as described earlier, with primer pairs 338 bf/517 ur and 344 af/517 ur, respectively, (final primer concentration: 300 nM; Probst et al., 2013). The primer sequences are as follows: Primer 338 bf (5^′^→ 3^′^): ACTCCTACGGGAGGCAGCAG (El Fantroussi et al., 1999), primer 517 ur (5^′^→ 3^′^): GWATTACCGCGGCKGCTG (Amann et al., 1995), primer 344 af (5^′^→ 3^′^): ACGGGGYGCAGCAGGCGCGA (Raskin et al., 1994). For each of the four biological replicates per olive sample, three technical qPCR replicates were carried out, using 1 μl of template DNA. Standard curves were developed from PCR products of the 16S rRNA gene of *Staphylococcus warneri* and *Nitrosopumilus maritimus*. The mean of the triplicates was calculated. The archaeal portion was calculated as part of the total 16S rRNA gene signatures retrieved (archaeal plus bacterial signals).

### ISOLATION OF ENDOPHYTES FROM OLIVES AND SCREENING FOR ANTAGONISTIC ACTIVITY TOWARDS *V. dahliae*

Bacterial isolates were obtained by plating aliquots of suspensions from plant materials on R2A (Difco, Detroit, MI, USA) and Kings B (containing 20 g proteose pepton, 15 ml glycerin, 1.5 g K_2_HPO_4_, 1.5 g MgSO_4_ × 7 H_2_O, 20 g agar per liter). The antagonistic activity of randomly selected isolates displaying morphologically distinct colonies (five to seven isolates each olive cultivar) towards the soilborne pathogen *V. dahliae* V25 was assessed by dual culture *in vitro* assay (Berg et al., 2005). Representative antagonistic isolates were characterized by BOX fingerprinting and partial 16S rRNA sequencing as described by Berg et al. (2005).

## RESULTS

### COMPOSITION OF ENDOPHYTIC MICROBIAL COMMUNITIES IN OLIVE TREES

**FIGURE 1:**
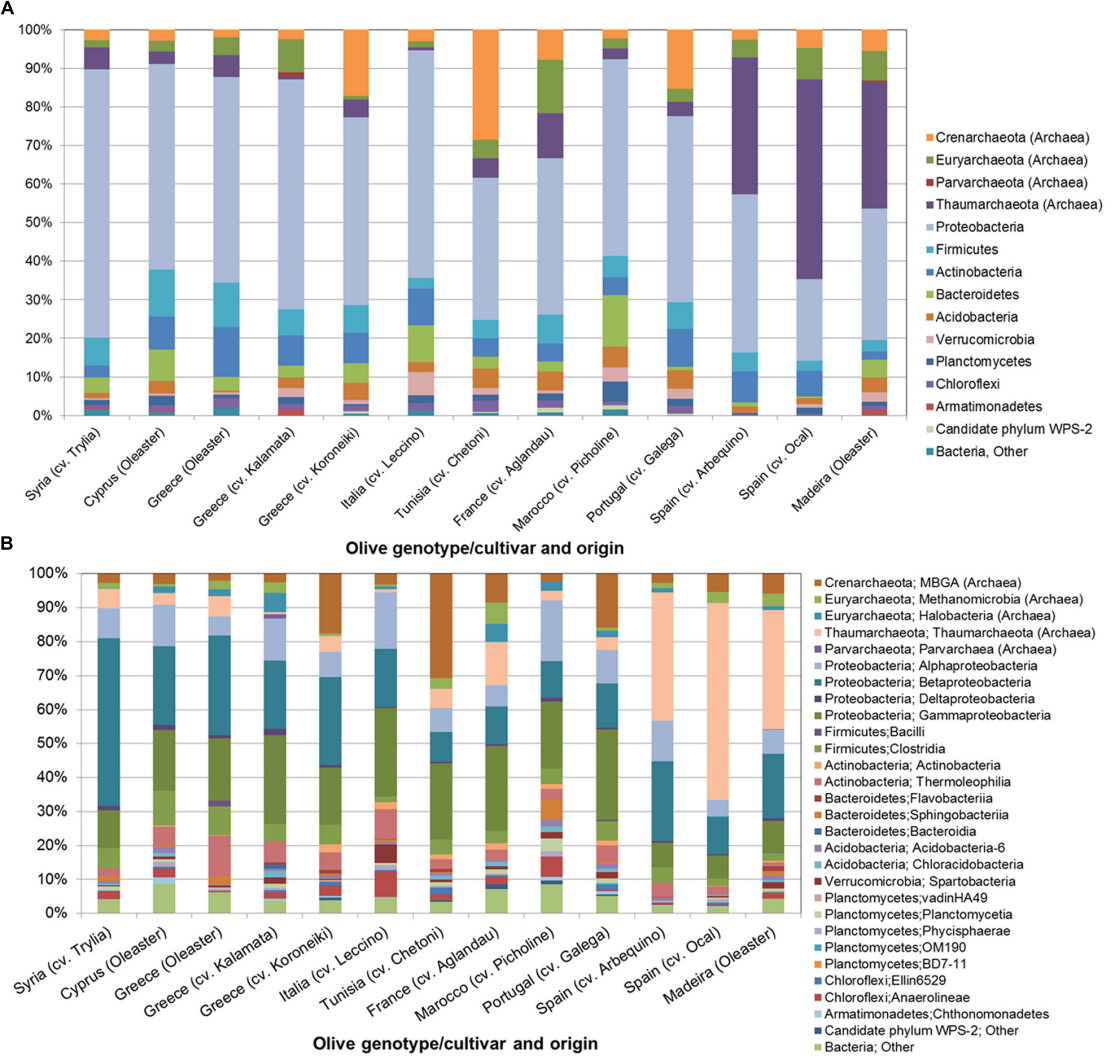
**Structure of the microbial communities in the endosphere of different olive cultivars revealed by Illumina MiSeq 16S rRNA gene amplicon sequencing at phylum **(A)** and class level **(B)****.

**FIGURE 2:**
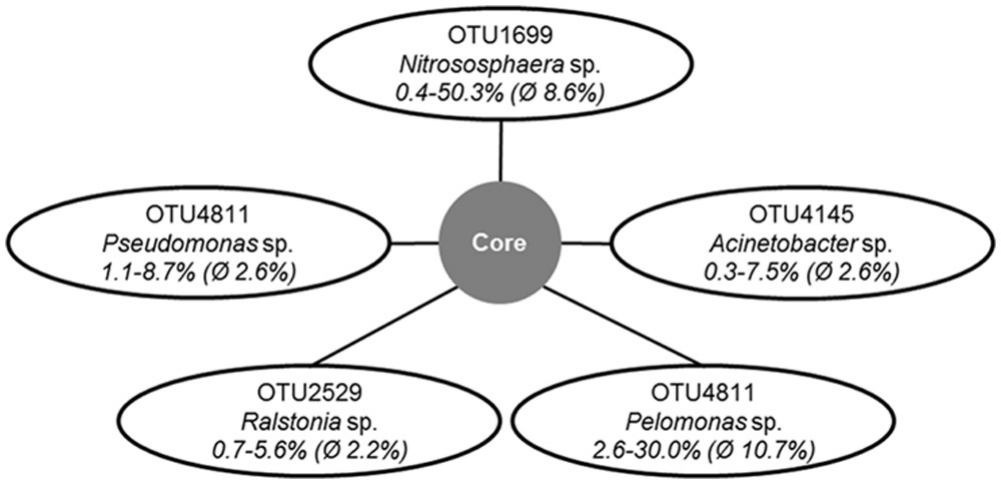
**Operational taxonomic units (OTUs) and their taxonomic affiliations representing the putative core microbiome in the endospheres of all studied olive samples.** The numbers indicate the maximum, minimum and average of relative abundances of the respective OTU throughout the read libraries.

The number of reads obtained by amplicon sequencing ranged from 210 to 1583 per olive cultivar (Supplementary Table S1). Based on the taxonomic classification of representative sequences from all OTUs, the composition of bacterial communities was revealed at phylum and class level (**Figures 1A,B**). Although PCR primers targeting eubacterial 16S rRNA genes were applied, a notable number of reads was assigned to the archaeal domain. Among all analyzed olive genotypes, the bacterial phylum *Proteobacteria* (21.3–69.6%) and the archaeal phyla *Thaumarchaeota* (0.6–51.7%) and *Crenarchaeota* (1.9–28.6%) predominated. Less abundant taxa that were detected in all samples belonged to *Firmicutes* (2.5–11.0%), *Euryarchaeota* (1.0–13.7%), and *Bacteroidetes* (0.4–13.4%). At class level all microbiomes contained representatives of *Alpha*-, *Beta*- ,and *Gammaproteobacteria* (4.9–17.9%, 8.8–49.4% and 6.7–26.6%), the archaeal classes *Thaumarchaeota* (0.6–58.1%) and MBG group A (2.0–30.7%), and *Bacilli* (1.8–10.3%). Out of 1,595 detected OTUs five OTUs were shared by all olive cultivars representing 9.8 to 61.0% of respective read counts (**Figure 2**). The putative core microbiome consisted of the betaproteobacterial *Pelomonas* sp. (10.7%) and *Ralstonia* sp. (2.2%), the thaumarchaeal candidate genus *Nitrososphaera* (8.6%), and the gammaproteobacterial *Pseudomonas* sp. (2.6%) and *Actinobacter* sp. (2.6%).

**FIGURE 3:**
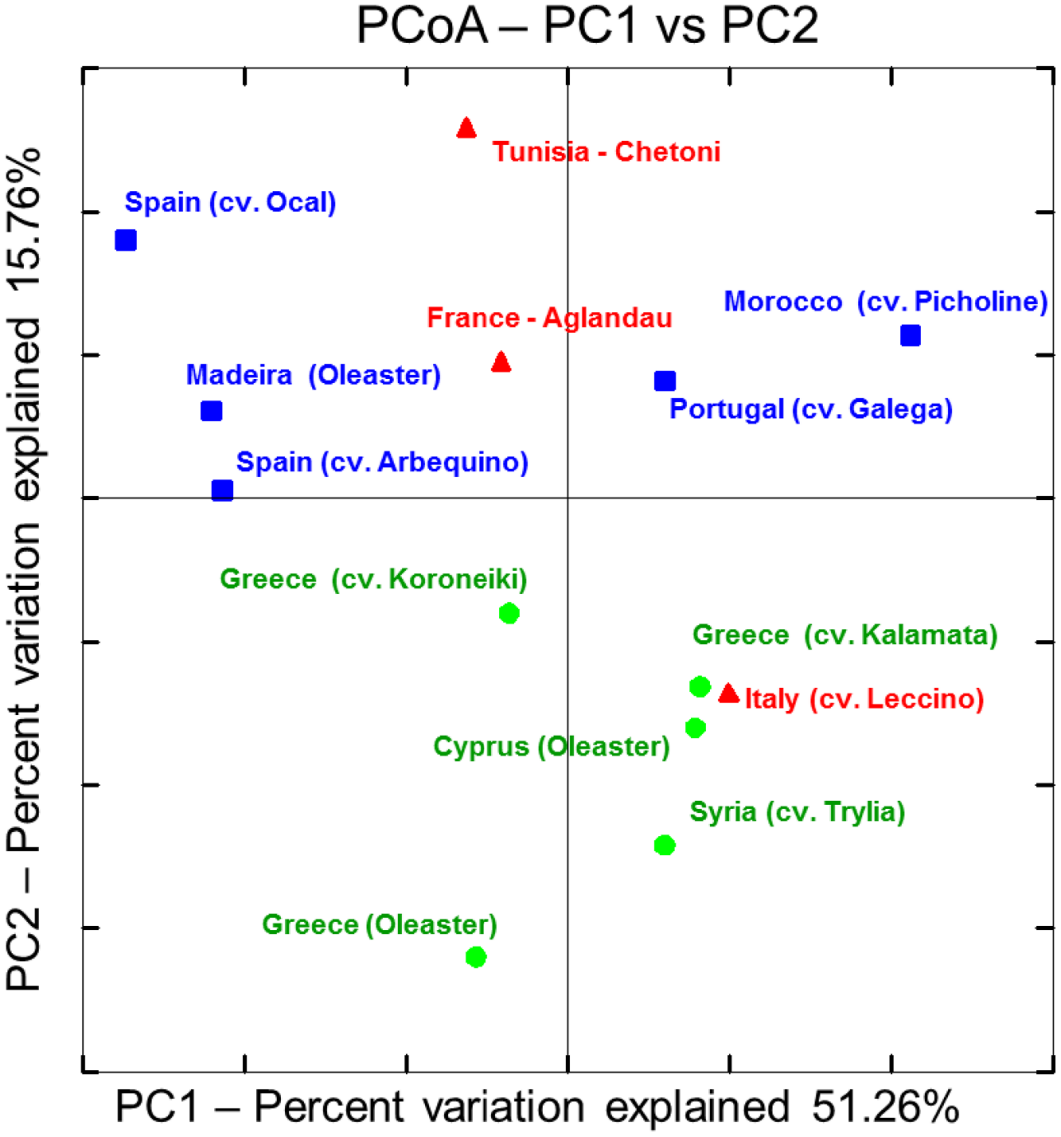
**Principal Coordinate Analysis (PCoA) plot deduced from weighted normalized UniFrac distance matrix calculated from OTU distribution obtained from 16S amplicon sequencing using Illumina’s MiSeq platform: Olive cultivar accessions were classified into three main geographical regions.** 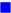 – Western Mediterranean. 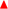 – Central Mediterranean. 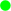 – Eastern Mediterranean.

Apart from commonalities, the analysis of the microbial endophytic communities indicated a high degree of cultivar and regional specificity. PCoA plot deduced from the distance matrix calculated by the weighted normalized UniFrac algorithm using phylogenetic information demonstrates a general clustering according to the geographic or cultural origin (**Figure 3**). Whereas the Western and Eastern olives are clearly distinguishable, the samples from the central Mediterranean basin (Tunisia, Italy, and France) were more similar to western (in case of Tunisia and France) or to eastern (Italy) olive cultivars. The exception could be explained by ancestors from a different region. The communities of wild olives sampled in Cyprus and Greece grouped closely within the cultivars originated from the same region. The microbiome of the oleaster from Madeira shares the most similarity to the olives from Spain, suggesting a cultural relationship. Analysis of the community composition measured by the UniFrac distances between the three regional groups showed that microbiomes from Western Mediterranean olives differed significantly from those of eastern cultivars and oleasters (*P* = 0.03), whereas there was no statistically significant differences between observed UniFrac distances from central and eastern olive groups or between central and western olive clusters.

**FIGURE 4:**
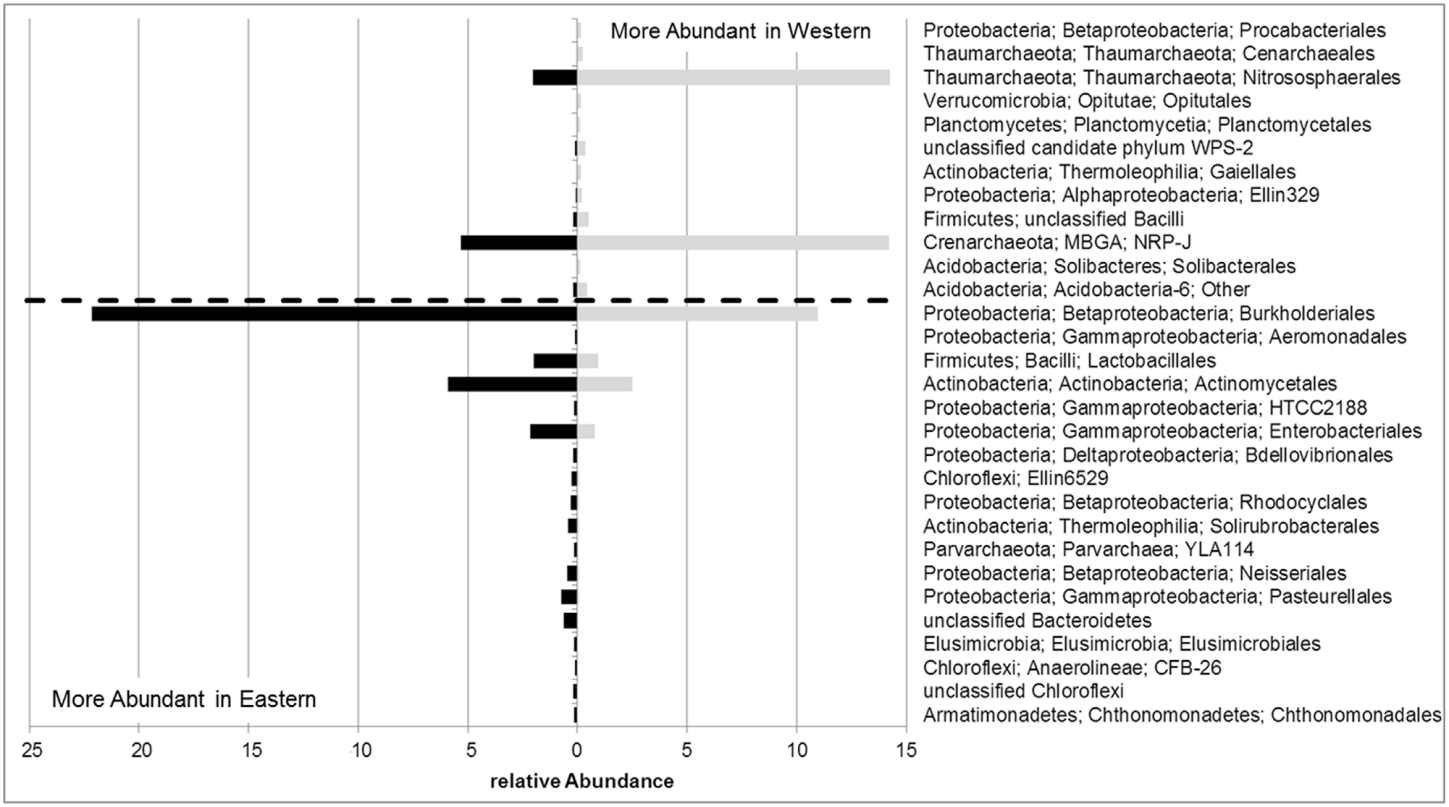
**Relative abundances of bacterial and archaeal orders with at least twofold differences in the endosphere of olive trees from eastern or western Mediterranean regions**.

The divergence of the microbial communities of olives from certain regions may be explained by variable abundances as well as by the presence and absence of particular taxonomic groups. **Figure 4** illustrates bacterial and archaeal orders identified in eastern and western olive trees with different relative abundances at a ratio higher than two. The bacterial orders *Chthonomonadales*, *Chloroflexi,* the candidate order CFB-26 and *Elusimicrobiales* were found exclusively in olive cultivars or oleasters originating in eastern Mediterranean regions. Among the most abundant eastern olive orders, *Burkholderiales* (2.0x), *Lactobacillales* (2.1x), *Actinomycetales* (2.4x) and *Enterobacteriales* (2.7x) were observed to be in increased numbers. In contrast, mainly archaeal orders were found in western olives. Here, *Crenarchaeales* were found exclusively, and the orders *Nitrososphaerales* (7.0x) and *Crenarchaeota* candidate order NRP-J (2.7x) were more dominant.

### ARCHAEAL POPULATION DENSITY AND COMMUNITY STRUCTURE

**FIGURE 5:**
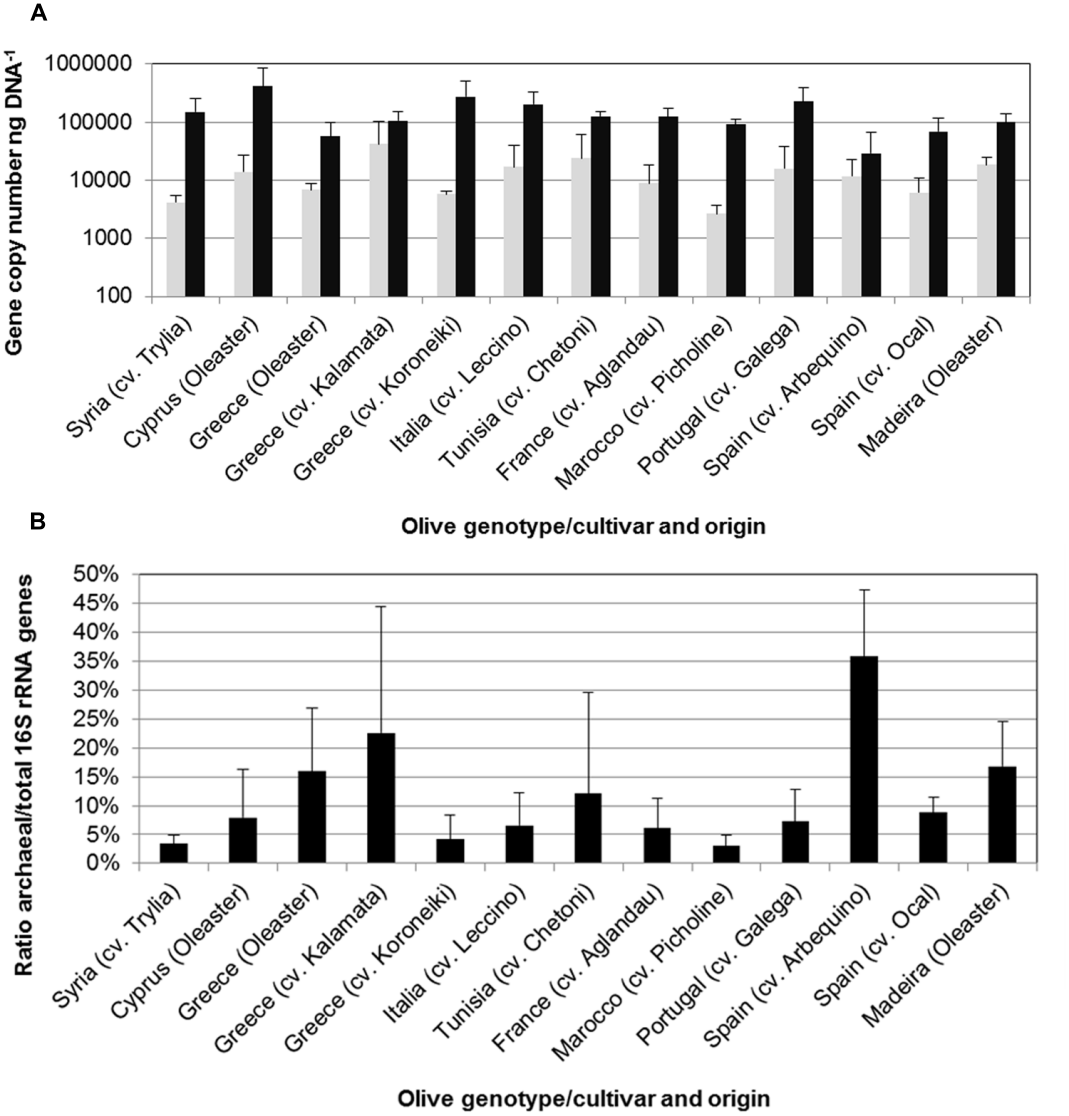
**Number of archaeal (gray bars) and total prokaryotic (black bars) 16S rRNA copies per ng template DNA **(A)** and proportion of archaeal 16S rRNA of total prokaryotic 16S rRNA gene copies **(B)** determined by quantitative real-time PCR (qPCR).** Error bars indicate confidence intervals at *P* = 0.05.

**FIGURE 6:**
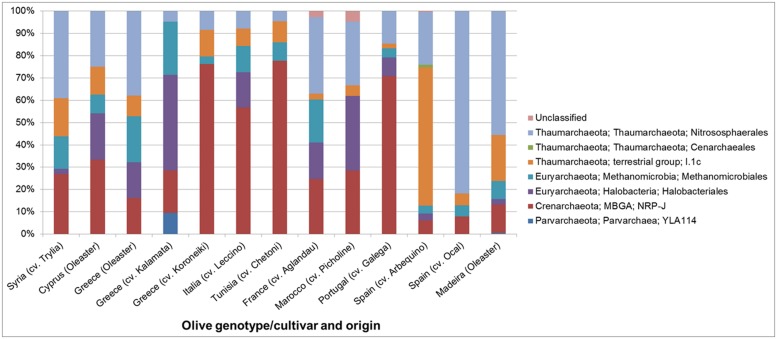
**Structure of the archaeal communities in the endosphere of different olive cultivars at order level revealed by Illumina MiSeq 16S rRNA gene amplicon sequencing**.

The structure and abundances of cultivars were analyzed in more detail because they appeared to be particularly well colonized by Archaea. The population density was quantified by qPCR (**Figure 5**), reaching up to 4 × 10^4^ copies per ng template DNA. 35.8% of all microbial 16S rRNA gene copies detected in the Spanish cultivar Ocal where found to be archaeal. It should be noted that because the eubacterial primers used also target plastid DNA, the relative abundances are likely to be higher than indicated by these measurements. The analysis of the amplicon library revealed a high proportion of endophytic Archaea in olive leaf tissues which account for 5.3 to 67.3% of total reads. The majority of archaeal reads were assigned to the phylum *Euryarchaeota* represented by the orders *Halobacteriales* and *Methanomicrobiales*, the phylum *Thaumarchaeota* with representatives in *Nitrososphaerales* and soil group I.1.b (*Nitrososphaera*) and I.1.c, and the *Crenarchaeota* candidate order NRP-J (**Figure 6**).

### ANTAGONISTIC ACTIVITY OF ENDOPHYTIC BACTERIAL STRAINS FROM OLIVE TREES

To assess the antagonistic potential of endophytes against *V. dahliae,* bacteria were isolated and tested on their *in vitro* antagonistic activity. The culturable bacterial population was 1.9 × 10^5^ colony forming units (CFU) g^-1^ fw^-1^ on average without any statistically significant differences between wild and cultivated olives (data not shown). Altogether, 80 randomly isolated strains were investigated regarding their antagonistic activity against the pathogen, from which 11.3% showed high antagonistic potential. Although bacteria were isolated from olive trees from different regions, those with high antagonistic activity showed highly similar fingerprints (analyzed by BOX patterns) suggesting that they belong to a similar genotype. The 16S rRNA gene of one representative strain from nine positively tested strains and was therefore sequenced and assigned to *Bacillus amyloliquefaciens* by blastn analysis (closest match: NR_116022.1, 99% identity).

## DISCUSSION

The structure of the endophytic microbiome of the 10 different olive cultivars correlated with their (breeding) geographical origin and was confirmed by the similarity of their microbiome structures shown by the nine wild oleasters from each region. In contrast, the function – we analyzed the antagonistic activity of endophytic isolates against the pathogenic fungus *V. dahliae* – was derived from the same bacterial genus *Bacillus*. Here, no impact of the region or breeding history was found. Moreover, *B. amyloliquefaciens* strains isolated from different cultivars and regions showed similar molecular fingerprints which suggested a close functional relationship.

We confirmed our hypothesis that olive trees and their endophytic microbiome can be divided at least into the major regions “Eastern” and “Western,” whereas the microbial populations of the three “Central” cultivars resemble those of one the other two zones. The microbial communities of the wild olives and of the cultivars from the corresponding regions were closely related. A high level of similarity between the microbial composition of wild trees from Cyprus and Greece and the cultivars with western origins were found. Additionally, the wild trees from Madeira possessed endophytes that were similar to the cultivars Arbequino and Ocal (Spain). These results show that compared to the influence of the olive genotype the prevailing soil and climate conditions at the sampling sites and the geographical distances of 1000s of kilometers have a negligible effect on the endophytic communities in leaves. Redford et al. (2010) found similar results by studying the phyllosphere of the *ponderosa* pine; however, several studies described the influence of environmental conditions on endophytic communities (Rosenblueth and Martínez-Romero, 2006). The abundance of the bacterial community depends on the age of the leaves and the endophytic diversity and was related to leaf traits of the tropical plant species *Coccoloba cereifera* (Sanchez-Azofeifa et al., 2012). Because the olive belongs to the evergreen tree species, the endophytic microbial diversity may be stable over longer periods of time.

Endophytes can promote the growth of plants and/or suppress phytopathogens (Backman and Sikora, 2008; Hardoim et al., 2008); however, the antagonistic part of the microbiome in this study was highly similar for all investigated genotypes. Only members of the *Firmicutes* group were found which are well-known as potent antagonists and biocontrol agents (Emmert and Handelsman, 1999). Molecular fingerprints and amplicon libraries confirmed the highly similar structure for all *Firmicutes* (data not shown). In amplicon libraries they present a proportion of less than 5%. The low proportion of potential antagonists within the culturable bacterial endophytes may be one reason for the high susceptibility of olive trees to *V. dahlia*e. *B. amyloliquefaciens* has been identified as the most important antagonistic species within the genus *Bacillus* which is well-known as a biological control agent (Marten et al., 2000; Kloepper et al., 2004) and a good colonizer of the olive rizosphere and rhizoplane (Aranda et al., 2011). To date, biological control approaches against *V. dahliae* in olive have targeted Gram-negative antagonists such as *Pseudomonas* and *Serratia* (Mercado-Blanco et al., 2004; Prieto and Mercado-Blanco, 2008; Prieto et al., 2009). Our results suggest that Gram-positive bacteria such as *Bacillus* from oleasters may also be an interesting option for biocontrol (Aranda et al., 2011).

The high proportion of archaeal 16S rRNA genes found in the endosphere of olive trees was the most interesting finding from our study. In a recent study (Cáliz et al., under revision) showed that rhizosphere of olive cultivars in southern Spain is mainly colonized by members of archaea belonging to 1.1b *Thaumarchaeota* (soil crenarchaeota group) closely related to the genus *Nitrososphaera*, with much less numbers of *Euryarchaeota* of the groups *Halobacteria, Methanomicrobia*, and *Thermoplasmata* indicating that olive select specific groups of Archaea as endophytes or that only specific groups of Archaea are adapted to live within olive tissues. Earlier studies on other plants indicate internal tissue colonization by members of Archaea*,* e.g., in *Phragmites australis* and *Coffea arabica* (Ma et al., 2013; Oliveira et al., 2013), and give comparably low numbers for their abundance (Redford et al., 2010; Finkel et al., 2011; Knief et al., 2012). In this study, we proof the presence of archaeal signatures in endophytic microbial communities from olive leafs, and propose a larger role of these microbes therein. *Thaumarchaeota* have been described as significant component in soil microbial community and even in the human skin microbiome (Probst et al., 2013). Due to their ammonia-oxidizing capability, they influence the local ammonia-availability and pH, which could help to defend pathogenic microorganisms and to maintain the healthy (endophytic) microbiome. Although the function of these Archaea remains speculative, this finding suggests the existence of an undiscovered action/mechanism that is essential to a more in-depth understanding of plant-archaea and human-archaea interactions.

## Conflict of Interest Statement

The authors declare that the research was conducted in the absence of any commercial or financial relationships that could be construed as a potential conflict of interest.
