# Supplementary material for: Plant genotype-specific archaeal and bacterial endophytes but similar Bacillus antagonists colonize Mediterranean olive trees
Source: Front Microbiol. 2015 Mar 3;6:138. doi: 10.3389/fmicb.2015.00138 (PMC4347506; doi:10.3389/fmicb.2015.00138)
Supplement: Supplementary file 1 [file table_1.pdf]

Supplementary Table 1: Number of reads per sample in course of data processing. Prior to quality filtering raw reads from the biological replicates were joined.

**Sample name**	**Olive cultivar (origin)**	**Replicate**	**Number of raw reads**	**Accession no. Sequence Read Archive (SRA, NCBI)**	**Number of quality reads**	**Number of reads after excluding plant plastid-derived reads**
SP3	Arbequino (Spain)	1	63,026			
		2	17,152			
		3	13,220			
		**Sum**	**93,398**	SRR1781607	72,800	1,583
SP5	Ocal (Spain)	1	109,325			
		2	47,994			
		3	93,325			
		**Sum**	**250,644**	SRR1781712	214,760	705
I2	Leccino (Italy)	1	54,316			
		2	77,720			
		3	78,790			
		**Sum**	**210,826**	SRR1781720	184,324	998
GR1	Koroneiki (Greece)	1	61,732			
		2	88,878			
		3	22,909			
		**Sum**	**173,519**	SRR1781736	155,490	320
GR2	Kalamata (Greece)	1	88,194			
		2	59,146			
		3	51,921			
		**Sum**	**199,261**	SRR1781767	168,378	210
TUN1	Chétoni (Tunesia)	1	97,612			
		2	104,814			
		3	88,720			
		**Sum**	**291,146**	SRR1781768	243,741	550
SI1	Trylia	1	72,028			
		2	77,835			
		3	89,262			
		**Sum**	**239,125**	SRR1781984	211,470	497
MO1	Picholine Marrocaine (Morocco)	1	96,852			
		2	85,352			
		3	109,840			
		**Sum**	**292,044**	SRR1781986	264,994	417
PO1	Galega (Portugal)	1	90,858			
		2	91,198			
		3	125,200			
		**Sum**	**307.256**	SRR1781987	268,713	292
FR1	Aglandau (France)	1	39,149			
		2	92,954			
		3	76,243			
		**Sum**	**208,346**	SRR1781988	182,079	278
CY	Oleaster (Cyprus)	1	95,967			
		2	65,062			
		3	92,225			
		**Sum**	**253,254**	SRR1781989	225,374	310
GR (wild)	Oleaster (Greece)	1	33,202			
		2	30,367			
		3	45,837			
		**Sum**	**109,406**	SRR1781990	95,836	716
M1	Oleaster (Madeira	1	75,616			
		2	25,052			
		3	76,027			
		**Sum**	**176,695**	SRR1782571	155,426	964
